# Relationships between pre‐, post‐, and inter‐ictal psychiatric symptoms in patients with epilepsy

**DOI:** 10.1002/pcn5.70369

**Published:** 2026-06-30

**Authors:** Shingo Yasumoto, Hiromichi Motooka, Yuji Ito, Masaya Mashimoto, Kenta Murotani, Ryota Ogata, Motohiro Ozone

**Affiliations:** ^1^ Department of Neuropsychiatry Kurume University School of Medicine Kurume Fukuoka Japan; ^2^ Department of Psychiatry Nishiwaki Hospital Nagasaki Nagasaki Japan; ^3^ School of Medical Technology Kurume University Kurume Fukuoka Japan; ^4^ Biostatistics Center Kurume University Kurume Fukuoka Japan; ^5^ Department of Biostatistics School of Medicine Kurume University Kurume Fukuoka Japan

**Keywords:** epilepsy, inter‐ictal, post‐ictal, pre‐ictal, psychiatric symptom

## Abstract

**Aim:**

Although pre‐ and post‐ictal psychiatric symptoms (PS) in patients with epilepsy are well known, the prevalences and pathophysiologies remain unclear. We investigated the prevalences and durations of pre‐ and post‐ictal PS, related factors, and associations between pre‐ and post‐ictal PS.

**Methods:**

In the Neuropsychiatry Department of Kurume University Hospital, patients with epilepsy were interviewed regarding pre‐ and post‐ictal PS. Multivariate logistic regression analyses were performed on clinical variables and pre‐ or post‐ictal PS. McNemar analyses were performed to clarify differences in the occurrence of pre‐ and post‐ictal PS.

**Results:**

Five percent of patients had only pre‐ictal PS, 29% had only post‐ictal PS, and 10% had both pre‐ and post‐ictal PS. The most common symptoms were depressive symptoms (25%) and anxiety symptoms (16%) in the post‐ictal period, and irritability symptoms (7%) and anxiety symptoms (5%) in the pre‐ictal period. The duration of PS was more than 1 day but less than 1 week in many patients. Post‐ictal PS showed a substantially higher ratio of a history or comorbidities of inter‐ictal psychiatric disorders (IPD). In addition, post‐ictal psychotic symptoms and post‐ictal irritability symptoms were significantly associated with psychosis in IPD. Furthermore, while the incidence of PS was significantly higher with post‐ictal PS alone than with pre‐ictal PS, cases with pre‐ictal PS had a significantly higher rate of post‐ictal PS.

**Conclusion:**

Post‐ictal PS appeared relevant to IPD, and pre‐ictal PS was associated with the expression of post‐ictal PS.

## INTRODUCTION

People with epilepsy (PWE) often experience various psychiatric symptoms (PS), such as anxiety, agitation, depression, manic states, and psychotic symptoms, which can be an issue. PS in PWE are classified according to the temporal relationship to epileptic seizures. Inter‐ictal PS are unrelated to the occurrence of seizures, but peri‐ictal (pre‐ or post‐ictal) PS occur around seizures and have been recognized for some time. Prodromal symptoms can occur hours to days before a seizure, differing from focal aware seizures (FAS), which occur for seconds to minutes at the beginning of a seizure. Prodromal symptoms in epilepsy are those seen during the pre‐ictal period. The International League Against Epilepsy (ILAE) defines prodromal symptoms as follows: “A pre‐ictal phenomenon. A subjective or objective clinical alteration (e.g., ill‐localized sensation or agitation) that heralds the onset of an epileptic seizure but does not form part of it.”^1^ According to this definition, prodromal symptoms are distinct from epileptic seizures, that is, FAS. Prodromal symptoms often manifest as PS, but may also appear as symptoms such as speech disorders, headaches, tremors, and nausea.^2^ Although the concept of FAS is useful in identifying the focus of seizure onset and is considered essential by clinicians, prodromal symptoms are not regarded as valuable and have received little attention. Historically, prodromal symptoms have been recognized since at least the time of Hippocrates, thousands of years ago.^3^ Gowers^4^ in the 19th century and Kraepelin^5^ in the 20th century also referred to phenomena occurring before seizures. Since then, however, little research has been conducted into the clinical experience of prodromal symptoms.^6^ The most common prodromal symptoms include funny feelings, confusion, irritability, and anxiety.^2^

PS also often occur after epileptic seizures. Post‐ictal psychosis can manifest as delusion and hallucination, often accompanied by psychomotor excitement and severe aggression, and is well‐known in current clinical practice as a serious clinical problem among PWE. In the 19th century, Jackson reported behavioral changes after seizures.^7^ He believed that after a seizure, high‐phase functionality was dismantled and the lower phase became active, producing an abnormal psychotic‐like mental state. Post‐ictal psychosis was conceptualized by Logsdail and Toone in the 20th century as a well‐established type of psychosis in epilepsy, and its clinical presentation was summarized.^8^ However, little attention has been paid to post‐ictal PS such as depression and anxiety, other than highly abnormal mental states such as psychosis and confusion. In a previous study, PS developed after seizure in 74% of patients. The most common post‐ictal PS were depression and anxiety, ranging in duration from a few minutes to several days.^9^

In this way, although a variety of PS have been reported in the peri‐ictal period, the symptoms may be clinically overlooked or mistakenly regarded as PS unrelated to seizures. In addition, a pattern of comorbid peri‐ and inter‐ictal PS and a pattern of inter‐ictal PS exacerbated in the peri‐ictal period have also been reported, suggesting a relationship between peri‐ictal PS and inter‐ictal PS.^9^, ^10^

Further, some patients clinically exhibit PS extending from the pre‐ictal to post‐ictal periods, suggesting that a continuum of functional alterations may occur before and after the seizure. However, to the best of our knowledge, the relationship between pre‐ and post‐ictal PS has not been investigated, and whether these phenomena represent independent entities or share a common pathophysiological basis remains unclear.

From a therapeutic perspective, peri‐ictal PS can be expected to improve with appropriate seizure management. Distinguishing peri‐ictal PS from PS unrelated to seizures is therefore of substantial clinical importance.

In this study, we investigated peri‐ictal PS in a broad sense, encompassing not only psychotic symptoms but also related psychopathological manifestations. Specifically, we examined the prevalence and characteristics of peri‐ictal PS in patients with epilepsy, analyzed factors associated with peri‐ictal PS, and analyzed the relationship and differences between pre‐ and post‐ictal PS.

## METHODS

### Patients and psychiatric assessments

Patients were recruited in the Neuropsychiatry Department of Kurume University Hospital between December 2019 and December 2021. In total, 290 patients over 16 years old and with a definite diagnosis of epilepsy were enrolled for the study. The regional Scientific Ethics Committee approved the study (approval no. 19165, date of approval: December 6, 2019), and informed consent was obtained from all patients and/or their family members. Patients with psychogenic non‐epileptic seizures were excluded.

**Figure 1:**
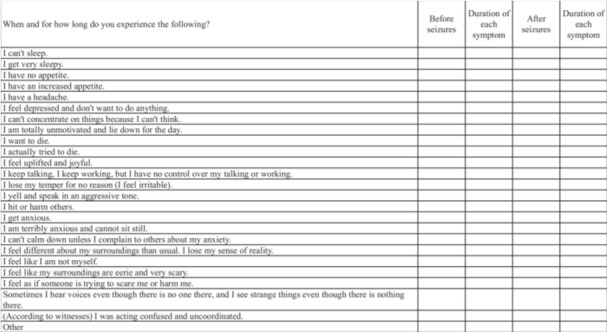
Peri‐ictal psychiatric symptoms (PS) questionnaire for the patient.

**Figure 2:**
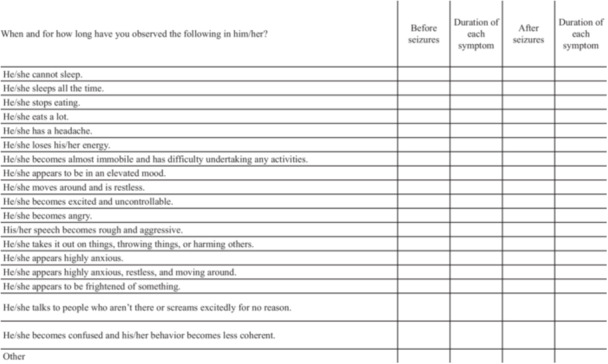
Peri‐ictal psychiatric symptoms (PS) questionnaire for family members of the patient.

Patients, their family members, and caregivers were interviewed by the attending psychiatrists, all of whom were certified psychiatrists of the Japanese Society of Psychiatry and Neurology and epileptologists of the Japan Epilepsy Society (S.Y., H.M., Y.I., and M.M.) to determine whether they had experienced any changes in subjective mental state or objective behaviors that were different from their usual state in the week before or after the seizure. If such symptoms were identified, we considered PS as present, and the duration of PS was determined using a questionnaire (Figures 1 and 2). The questionnaire items were developed based on a comprehensive review of the literature and expert consensus. Two board‐certified psychiatrists and epileptologists (S.Y. and H.M.) selected the items to ensure clinical relevance and content validity for evaluating peri‐ictal PS.

We evaluated PS before and after past habitual seizures. When PS appeared between seizures, we focused on the time between symptom onset and seizure occurrence to identify the seizure most likely associated with the PS and then classified the symptoms as pre‐ictal or post‐ictal relative to the seizure closest in time. Specifically, PS emerging between Seizures 1 and 2 were classified as post‐ictal if symptom onset was closer to Seizure 1 and as pre‐ictal if onset was closer to Seizure 2. In cases of seizure clusters, the duration of the cluster was treated as a single unit, with the period before the cluster designated as the pre‐ictal period. The period during and after the cluster was then designated as the post‐ictal period.

To obtain a report as accurate as possible in terms of peri‐ictal PS, we excluded patients who had not experienced any seizures for more than 1 year. Regarding objective behavioral changes in cases involving severe mental retardation and dementia, distinguishing whether behavioral changes were due to mood changes or disturbances in consciousness and cognition is difficult, so only subjective symptoms were included in the analysis, and severe mental retardation and dementia were excluded. Of the 290 cases, 47 showed objective behavioral changes rather than subjective ones, and 100 cases had been seizure‐free for at least 1 year. Of these, 10 duplicate cases demonstrated both objective behavioral changes and seizure‐free status for at least 1 year. As a result, 137 cases were excluded, and the remaining 153 cases were included in the analysis.

This study classified PS before and after seizures into the following five categories based on established clinical descriptions of peri‐ictal psychiatric phenomena. Specifically, the categories for pre‐ictal PS were derived from Besag and Vasey, while those for post‐ictal PS were based on the clinical characteristics reported by Kanner et al.^2^, ^9^:

- Anxiety symptoms.
- Depressive symptoms (characterized by depressed mood or hypobulia).
- Irritability symptoms (including irritability and aggressive behavior).
- Manic symptoms (such as mood elevation, hyperactivity, or pressured speech).
- Psychotic symptoms (including delusional ideas and hallucinations).

**Figure 3:**
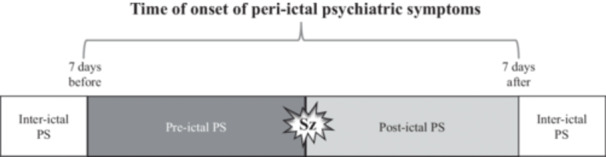
Definition of pre‐ and post‐ictal psychiatric symptoms (PS). Five categories are based on these findings: anxiety symptoms; depressive symptoms (characterized by depressed mood or hypobulia); irritability symptoms (including irritability and aggressive behavior); manic symptoms (such as mood elevation, hyperactivity, or pressured speech); and psychotic symptoms (including delusional ideas and hallucinations).

Initially, we had classified six categories, including “confusion” alongside the other five categories. However, since “confusion” primarily refers to symptoms observed objectively and the scope of this study limited PS to subjective symptoms, “confusion” was excluded. We excluded any PS that occurred as a result of a known event (e.g., depression due to the end of a relationship), as these could be psychogenic reactions. This is because in these cases, PS is considered a precipitating phenomenon rather than a prodromal symptom. Symptoms such as headaches, insomnia, hypersomnia, and decreased or increased appetite listed in the questionnaire were considered as nonspecific symptoms that could accompany any subcategory of PS, and therefore were not classified into specific categories. Post‐ictal psychotic symptoms may be confused with post‐ictal confusion. However, unlike post‐ictal confusion, post‐ictal psychotic symptoms are characterized by distinct hallucinations and delusions, and patients retain some level of consciousness and are afterward able to recall their state at that time. Further, while confusion is observed immediately following a seizure, a psychotic state may be preceded by a symptom‐free period of 1–2 days, representing the so‐called lucid interval. Post‐ictal psychotic symptoms are distinguished from post‐ictal confusion based on the above. We also focused on the durations of PS. To distinguish between FAS and pre‐ictal PS, we defined pre‐ictal PS as those PS lasting more than 30 min and distinct from symptoms that typically last less than 1 min (i.e., FAS), which occur at the beginning of a seizure. In summary, pre‐ and post‐ictal PS were defined as shown in Figure 3.

In addition, clinical variables such as age, sex, age at epilepsy onset, diagnosis of epilepsy, type of seizure, past history, etiologic lesion, laterality of inter‐ictal discharges (IID) on the electroencephalogram (EEG), and past history or comorbidity of inter‐ictal psychiatric disorders (IPD) were collected from medical records. The type of epilepsy was based on the ictal semiology, results from electroencephalography, and neuroimaging findings, according to the definitions proposed by the ILAE.^11^

IPD is a chronic or transient condition that is not temporally related to the seizures. In this study, medical records and examination findings were used to confirm the presence of IPD. Further, IPD was classified according to the *International Statistical Classification of Diseases and Related Health Problems, 10th Revision* (ICD‐10) criteria for Mental and Behavioral Disorders,^12^ as follows:

- **Psychosis** was defined as meeting the criteria for organic hallucinosis (F06.0), organic catatonic disorder (F06.1), or organic delusional disorder (F06.2). Psychotic symptoms, including hallucinations, delusions, thought disorder, or catatonic symptoms, such as psychomotor excitement or stupor, were required for diagnosis.
- **Depression and mania** were defined as meeting the criteria for organic mood disorder (F06.3), characterized primarily by mood fluctuations. Mood disturbances occurring prior to the onset of epilepsy were classified as a depressive episode (F32) or bipolar disorder (F31).
- **Anxiety** was defined as meeting the criteria for organic anxiety disorder (F06.4), presenting with clinical features consistent with panic disorder and generalized anxiety disorder.
- **Irritability**, mainly characterized by aggression and anger, was classified as an unspecified mental disorder due to brain damage and dysfunction, as well as to physical disease (F06.9).

Since none of the cases in this study presented with schizophrenia or anxiety disorders prior to the onset of epilepsy, none of the subjects were classified under F2 or F4.

In some patients with IPD, multiple diagnostic categories were present.

Regarding the coexistence of inter‐ and post‐ictal PS, when these symptoms occurred at different times, both types of PS were considered to coexist in the same patient. On the other hand, post‐ictal PS that occurred during a period of ongoing chronic inter‐ictal PS were classified as post‐ictal PS if the patient exhibited PS that differed from those usually observed, such as if a patient with chronic psychotic symptoms developed a depressive state after a seizure. Further, during the period of chronic PS, if worsening behaviors such as psychomotor agitation, violent behavior, or suicidal attempts were observed following a seizure, this was judged to represent a complication of post‐ictal PS.

### Statistical analysis

Fisher's exact test was performed to evaluate differences in the rates of pre‐ and post‐ictal PS. Multivariate logistic regression analysis was performed to evaluate clinical variables associated with pre‐ or post‐ictal PS. Associations between each subgroup and clinical variables were also analyzed. Variables showing values of *p* < 0.1 in univariate analyses were considered as candidate variables for multivariate logistic regression analysis. Among the candidate variables, those representing similar or hierarchical constructs were not included simultaneously in the same model to avoid conceptual overlap and multicollinearity. In such cases, variables with greater clinical relevance and interpretability were selected. The significance level for multivariate analysis was set at *p* < 0.05. Differences in the occurrence of pre‐ and post‐ictal PS were evaluated using McNemar's test. In addition, the association between pre‐ and post‐ictal PS was assessed using odds ratios (ORs) with 95% confidence intervals (CIs) derived from contingency tables. All analyses were conducted using R version 4.2.1.

## RESULTS

### Clinical data of patients

Table 1 shows the clinical data of patients. In total, 153 patients (male, *n* = 82; female, *n* = 71) were included in this study. Mean age was 42.5 ± 15.6 years, and mean age at the onset of epilepsy was 18.8 ± 16.4 years. The underlying diagnosis was focal epilepsy in 130 patients, idiopathic generalized epilepsy in 15, combined generalized and focal epilepsy in 2, and benign adult familial myoclonic epilepsy in 2. Of the 130 patients with focal epilepsy, 48 had temporal lobe epilepsy (TLE). Seventy‐six patients had IPD, which was classified into anxiety, depression, irritability, psychosis, or mania (as mentioned above).

**Table 1:** Clinical data of patients.

Total sample		153
Sex (male/female)		82/71
Age, years (mean ± SD)		42.5 ± 15.6
Age at onset of epilepsy, years (mean ± SD)		18.8 ± 16.4
Diagnosis of epilepsy		
Focal	Temporal	48
	Extratemporal	82
Idiopathic generalized		15
Combined generalized and focal		2
Others		2
Unknown		4
Type of seizure		
Focal aware		84
Focal impaired awareness		95
Tonic‐clonic		113
Past history		
Febrile convulsion		21
Perinatal abnormality		19
Encephalitis		15
Head trauma		8
Etiology		
Medial temporal sclerosis		16
Cortical dysplasia		4
Brain tumor		3
Laterality of inter‐ictal discharges on EEG		
Normal		25
Right		29
Left		27
Bilateral		49
Generalized		23
History or comorbidity of inter‐ictal psychiatric disorders		76
Anxiety (F06.4)		14
Depression (F06.3, F31, F32)		27
Irritability (F06.9)		17
Psychosis (F06.0, F06.1, F06.2)		25
Mania (F06.3, F31)		2

Abbreviation: EEG, electroencephalogram.

### Prevalence of peri‐ictal PS

**Figure 4:**
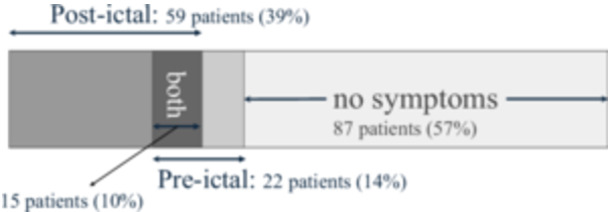
Number and percentage of patients with pre‐ictal psychiatric symptoms (PS), post‐ictal PS, or no peri‐ictal PS.

Table 2 shows the number of pre‐ and post‐ictal PS. Of the 153 patients, 22 (14%) reported pre‐ictal PS and 59 (39%) reported post‐ictal PS. Fifteen patients (10%) displayed both pre‐ and post‐ictal PS, 7 (5%) had pre‐ictal PS alone, and 44 (29%) had only post‐ictal PS. The remaining 87 patients (57%) had no symptoms either before or after seizures (Figure 4). As mentioned above, peri‐ictal PS were divided into five categories (anxiety symptoms, depressive symptoms, irritability symptoms, manic symptoms, and psychotic symptoms). Some patients showed PS in multiple categories. Concerning the number of categories of pre‐ictal PS shown, 19 patients had PS in one category, and the remaining 3 showed PS in two categories. As for categories of post‐ictal PS, 39 patients showed PS in one category, 16 showed PS in two categories, and the remaining 4 had PS in three categories. The most common category of pre‐ictal PS was irritability symptoms (10 patients, 7% of the 153 patients), followed by anxiety symptoms (8 patients, 5%). Among patients with post‐ictal PS, the most common category was depressive symptoms (39 patients, 25%), followed by anxiety symptoms (25 patients, 16%). Comparing pre‐ and post‐ictal PS, Fisher's exact test showed that post‐ictal PS were significantly more common than pre‐ictal PS in the total cohort, and in the subgroups of PS, anxiety symptoms, depressive symptoms, and psychotic symptoms were significantly more common post‐ictally than pre‐ictally.

**Table 2:** Number of pre‐ and post‐ictal psychiatric symptoms (PS).

	Pre‐ictal PS	Post‐ictal PS	*p*
Total PS	22 (14%)	59 (39%)	<0.0001
Anxiety symptoms	8 (5%)	25 (16%)	0.0027
Depressive symptoms	4 (3%)	39 (25%)	<0.0001
Irritability symptoms	10 (7%)	7 (5%)	0.619
Manic symptoms	2 (1%)	2 (1%)	1
Psychotic symptoms	1 (1%)	10 (7%)	0.0104

*Note*: Fisher's exact test shows a significant difference in rates of pre‐ and post‐ictal PS.

### Duration of PS

The duration of pre‐ictal PS was within 1 hour for 2 patients, between 1 hour and less than 1 day for 7, and between 1 day and less than 1 week for 13. Meanwhile, the duration of post‐ictal PS was within 1 hour for 10 patients, between 1 hour and less than 1 day for 13, between 1 day and less than 1 week for 30, and 1 week or longer for 6. Both pre‐ and post‐ictal PS predominantly lasted between 1 day and less than 1 week.

Among the six patients whose post‐ictal PS persisted for 1 week or longer, post‐ictal psychotic symptoms were present in half of them (three patients).

### Relationship between peri‐ictal PS and clinical variables

Regarding the relationship between peri‐ictal PS and clinical variables, including IPD, the supplementary table shows the results of univariate analyses (Supporting Information Table S1 for pre‐ictal PS and Supporting Information Table S2 for post‐ictal PS). Table 3 shows the results of multivariate logistic regression analyses conducted on the variables selected from these findings. Multivariate logistic regression analysis showed no significant associations with pre‐ictal PS. Conversely, post‐ictal PS were significantly associated with IPD (49% in IPD vs. 29% in non‐IPD groups; *p* = 0.021, OR 2.246, 95% CI 1.131–4.46) and younger age at epilepsy onset (15.1 vs. 21.2 years; *p* = 0.045, OR 0.975, 95% CI 0.951–0.999).

**Table 3:** Multivariate logistic regression analyses regarding relationship between pre‐ and post‐ictal psychiatric symptoms (PS) and the clinical variance.

Peri‐ictal PS		Univariate logistic regression			Multivariate logistic regression		
Post‐ictal PS	Clinical variables	OR	95% CI	*p*	OR	95% CI	*p*
	IPD	2.372	1.216–4.628	0.011	2.246	1.131–4.46	0.021*
	Sex	1.87	0.968–3.614	0.062	1.791	0.905–3.546	0.094
	Age at onset of epilepsy	0.974	0.951–0.997	0.03	0.975	0.951–0.999	0.045*

Post‐ictal anxiety		Univariate logistic regression			Multivariate logistic regression		
		OR	95% CI	*p*	OR	95% CI	*p*
	Psychosis in IPD	2.377	0.87–6.494	0.091	2.433	0.784–7.548	0.124
	Sex	2.36	0.97–5.737	0.058	2.244	0.854–5.895	0.101
	Cortical dysplasia	5.478	0.734–40.874	0.097	5.016	0.574–43.833	0.145
	Focal impaired awareness	0.384	0.16–0.919	0.032	0.215	0.075–0.614	0.004*
	Right side discharges on EEG	3.825	1.499–9.76	0.005	5.159	1.714–15.529	0.004*

Post‐ictal irritability		Univariate logistic regression			Multivariate logistic regression		
		OR	95% CI	*p*	OR	95% CI	*p*
	Irritability in IPD	7.071	1.435–34.854	0.016	8.26	0.859–79.414	0.067
	Psychosis in IPD	7.937	1.657–38.021	0.01	20.313	2.161–190.937	0.008*
	Age at onset of epilepsy	0.911	0.816–1.016	0.094	0.879	0.733–1.054	0.164
	Tonic‐conic	0.245	0.052–1.149	0.074	0.275	0.035–2.159	0.219
	Left side discharges on EEG	7.13	1.496–33.992	0.014	14.646	1.69–126.884	0.015*

Post‐ictal psychotic		Univariate logistic regression			Multivariate logistic regression		
		OR	95% CI	*p*	OR	95% CI	*p*
	Psychosis in IPD	6.15	1.632–23.175	0.007	5.767	1.493–22.274	0.011*
	Bilateral discharges on EEG	3.488	0.937–12.989	0.063	3.222	0.832–12.475	0.09

*Note*: Multivariate logistic regression analysis.

Abbreviations: CI, confidence interval; EEG, electroencephalogram; IPD, inter‐ictal psychiatric disorders; OR, odds ratio.

* *p* < 0.05.

In addition, relationships between each subgroup of peri‐ictal PS and clinical variables were analyzed. Post‐ictal anxiety symptoms were significantly associated with absence of focal impaired awareness seizures (FIAS) (12% in FIAS vs. 25% in non‐FIAS groups; *p* = 0.004, OR 0.215, 95% CI 0.075–0.614), and right‐side IID on EEG (34% in right‐side IID vs. 12% in non‐right‐side IID groups; *p* = 0.004, OR 5.159, 95% CI 1.714–15.529). Post‐ictal symptoms of irritability were significantly associated with left‐side IID on EEG (15% in left‐side IID vs. 2% in non‐left‐side IID groups; *p* = 0.015, OR 14.646, 95% CI 1.69–126.884), and psychosis in IPD (16% in psychosis in IPD vs. 2% in non‐psychosis in IPD groups; *p* = 0.008, OR 20.313, 95% CI 2.161–190.937). Post‐ictal psychotic symptoms were associated with psychosis in IPD (20% in psychosis in IPD vs. 4% in non‐psychosis in IPD groups; *p* = 0.011, OR 5.767, 95% CI 1.493–22.274). In this way, overall post‐ictal PS and multiple subgroups of post‐ictal PS were associated with IPD.

### The relationship and differences between pre‐ and post‐ictal PS

Fifteen patients exhibited both pre‐ and post‐ictal PS, whereas seven patients exhibited only pre‐ictal PS. Forty‐four patients exhibited only post‐ictal PS, and 87 patients showed no PS. McNemar's test showed significant asymmetry in the occurrence of PS before and after seizures (*p* < 0.0001) (Table 4). The number of cases with only post‐ictal PS was significantly higher than the number of cases with only pre‐ictal PS. Further, among cases with pre‐ictal PS, a high proportion (68%, 15/22 cases) also exhibited post‐ictal PS, whereas among cases without pre‐ictal PS, only 34% showed post‐ictal PS (44/131 cases). Patients who exhibited pre‐ictal PS displayed a higher risk of exhibiting post‐ictal PS compared to those who did not (OR 4.24, 95% CI 1.61–11.18).

**Table 4:** McNemar analysis showing significant asymmetry in the occurrence of pre‐ and post‐ictal psychiatric symptoms (PS).

	Post‐ictal PS	No post‐ictal PS
Pre‐ictal PS	15	7
No pre‐ictal PS	44	87

*Note*: McNemar test, *p* < 0.0001.

Of the 15 patients who exhibited both pre‐ and post‐ictal PS, 8 patients showed concordance in the categories of PS observed before and after seizures. Further, four patients demonstrated the same categories of PS even in IPD. In contrast, in the remaining seven patients, the categories of pre‐ictal PS differed from those of post‐ictal PS. The most frequent pattern was pre‐ictal anxiety symptoms and post‐ictal depressive symptoms (six cases), followed by pre‐ictal irritability symptoms and post‐ictal depressive symptoms (four cases), pre‐ictal anxiety symptoms and post‐ictal anxiety symptoms (four cases), and pre‐ictal irritability symptoms and post‐ictal anxiety symptoms (three cases).

## DISCUSSION

### Prevalence and duration of peri‐ictal PS

In our study, the prevalence of pre‐ictal PS was 14%, and the most common pre‐PS was irritability symptoms (7%), followed by anxiety symptoms (5%). A previous study described the rate of prodromal symptoms occurring with epileptic seizures as ranging from 2% to 87%.^2^ Besag and Vasey speculated that this discrepancy was due to methodological differences (such as the use of interviews or questionnaires), the strictness of the criteria applied for differentiating prodromal symptoms and FAS, and the inclusion or exclusion of objective prodromal symptoms in addition to subjective prodromal symptoms.^2^ In fact, previous studies reporting high‐frequency prodromal symptoms have not described a strict distinction from FAS.^6^, ^13^, ^14^, ^15^ Pre‐ictal PS lasting less than 30 min was excluded from the present study because of the difficulty in differentiating such symptoms from FAS.

Although the proportion of prodromal symptoms (39%) was relatively high in the report by Scaramelli et al., the authors stated that this may have been due to the inclusion of objective behavioral changes in addition to subjective symptoms.^16^ On the other hand, our study aimed to assess PS among prodromal symptoms, but only subjective symptoms were included because distinguishing whether behavioral changes in severe mental retardation and dementia were due to mood changes or disturbances in consciousness or cognition was difficult. Further, we excluded symptoms other than PS, such as speech disturbances, headache, tremor, and nausea. As a result, our reported incidence is likely lower than the incidence of prodromal symptoms as described by Scaramelli et al.

A meta‐analysis in a previous study found that prodromal symptoms mainly appeared as PS, and the most common symptom was funny feelings (10.4%), followed by confusion (9%), anxiety (8.6%), and irritability (7.7%).^2^ The high incidence of irritability (7%) and anxiety (5%) in our study was consistent with previous findings.^16^, ^17^ On the other hand, “funny feelings” was the most commonly reported prodromal symptom in previous studies. However, bias has been identified in the literature reporting this symptom.^13^, ^14^, ^17^ The majority of cases with funny feelings were reported in the same group,^13^, ^14^ observed at a high frequency of 62%, whereas the frequency of funny feelings was only 3% in the remaining report.^17^ Thus, “funny feelings” are not necessarily a symptom that is generally and widely recognized. Further, “funny feeling” refers to a sense of unease that is difficult to express in words, such as “something feels off” or “a strange sensation.” This sensation appears to refer to a more primitive and vague sensation rather than a distinct PS. As a result, we did not include this among PS in the present study.

In our study, the prevalence of post‐ictal PS was 39%. While few studies have investigated post‐ictal PS other than psychosis, a previous report by Kanner et al. identified 74% of patients with epilepsy as experiencing some PS during post‐ictal periods, including 43% with depression, 45% with anxiety, 7% with psychosis, and 22% with hypomania.^9^

The prevalence of post‐ictal PS was lower in our study than reported by Kanner et al.^9^ This may be due to differences in population and methodology. While Kanner et al. prospectively investigated patients with pharmacoresistant focal epilepsy followed at their Epilepsy Center who had undergone video‐EEG monitoring, our study included outpatients with overall epilepsy in a psychiatry department, and PS were identified retrospectively. However, patients in our study also showed a high ratio of depressive symptoms and anxiety symptoms, similar to the findings reported by Kanner et al.

Numerous reports have described post‐ictal psychotic symptoms, and a meta‐analysis^18^ reported a prevalence of 4% for post‐ictal psychotic symptoms, somewhat lower than the present results (7%). Although post‐ictal psychosis is generally extracted from patients presenting with severe symptoms such as psychomotor excitement, our study identified subjective psychotic symptoms, including mild cases unnoticed by others and those noticed but resolving quickly without causing significant problems. Such cases may usually be overlooked by physicians.

Many of our cases showed both pre‐ and post‐ictal PS extending from 1 day to less than 1 week in duration. Some studies have evaluated the duration of prodromal symptoms within 24 hours before seizures,^16^ while others have reported that onset can be seen as early as 1 week prior.^19^ We therefore also evaluated PS onset within the week prior to seizure onset, and many patients reported PS onset more than 1 day prior to seizure onset; it is possible that the evaluation of pre‐ictal PS onset within 24 hours omits PS.

In addition, the duration of post‐ictal PS has been reported as lasting from a few minutes to several days, with a median duration less than 24 hours.^9^ These durations tended to be somewhat shorter than in our cases. Some post‐ictal PS were prolonged, lasting more than a week in the present study. Half of these cases represented post‐ictal psychotic states, and 8 out of 10 cases (80%) presenting with post‐ictal psychotic states lasted for 1 day or longer. Post‐ictal psychosis commonly lasts several days, but can last for weeks according to previous studies,^20^ as in our study. In general, post‐ictal psychotic states represent a greater intensity of PS compared with other peri‐ictal PS. As a result, post‐ictal psychotic states are also speculated to typically require a longer period of recovery.

### Relationships between pre‐, post‐, and inter‐ictal PS

In our cases, post‐ictal PS were significantly related to IPD. In addition, post‐ictal psychotic symptoms and post‐ictal irritability symptoms were both associated with psychosis in IPD. In this manner, correlations between post‐ictal PS and IPD were suggested, particularly for irritability and psychotic symptoms.

Kanner et al. also described a relationship between post‐ictal PS and IPD, particularly for depression, anxiety, and psychosis.^9^, ^10^ While patients with both inter‐ and post‐ictal psychosis have been reported,^21^, ^22^ the psychotic symptoms were similar in both periods. We have previously reported a patient with both post‐ and inter‐ictal psychosis in whom each psychotic state presented as similar PS with similar changes in epileptic discharges on inter‐ictal EEG and in brain blood flow.^23^ Based on such studies, peri‐ and inter‐ictal PS may involve common mechanisms related to epileptic activities. On the other hand, Kanner et al. pointed to a pattern of post‐ictal exacerbation of IPD. We also observed such a pattern among our cases. Kanner et al. also noted that a history of IPD puts patients at risk of post‐ictal PS.^9^ Moreover, Adachi et al. speculated that patients with both inter‐ and post‐ictal psychosis are more vulnerable to developing psychotic symptoms.^22^ Patients with peri‐ictal PS might have some sort of psychiatric vulnerability, and seizures might precipitate PS.

Among cases that exhibited pre‐ictal PS, post‐ictal PS were observed in 68% of cases, representing a significantly higher incidence compared to cases that did not exhibit pre‐ictal PS. Pre‐ and post‐ictal PS have been described independently to date. Although few reports have examined both pre‐ and post‐ictal PS, Mula et al. described peri‐ictal dysphoric symptoms, reporting that 12% of all patients showed peri‐ictal dysphoric symptoms.^24^ Forty‐one percent of patients with peri‐ictal dysphoric symptoms showed PS before seizures, and 29% showed PS after seizures. However, PS in patients with both pre‐ and post‐ictal PS were not described.^24^ Our results suggest that PS in the peri‐ictal period are often seen both pre‐ and post‐ictally, and that a series of functional changes associated with seizures may have occurred from before to after seizures. In fact, when 15 patients with both pre‐ and post‐ictal PS were investigated in detail, the contents of PS in 8 patients were similar in both periods.

On the other hand, McNemar's test showed that cases with post‐ictal PS alone were significantly more frequent than cases with pre‐ictal PS alone. As shown in Table 2, when comparing pre‐ and post‐ictal PS, the prevalence of post‐ictal PS was significantly higher overall. In terms of subgroups for PS, anxiety symptoms, depressive symptoms, and psychotic symptoms were significantly more common after than before seizures. In addition, although no significant difference was seen, irritability symptoms were slightly more common with pre‐ictal PS than with post‐ictal PS. The fact that irritability was more prevalent before seizures than after was also consistent with the results reported by Mula et al.^24^ A difference thus existed between pre‐ and post‐ictal PS. Further, in cases with both pre‐ and post‐ictal PS, many patterns of different PS were seen before and after the seizure, such as pre‐ictal anxiety symptoms and post‐ictal depressive symptoms, and pre‐ictal irritability symptoms and post‐ictal depressive symptoms. If a patient presents with different PS before and after the seizure, different mechanisms could be at work before and after the seizure. For example, PS before seizures may reflect increased epileptic activity in the process leading up to seizure onset, while PS after seizures may reflect changes in epileptic activity due to whatever strong inhibitory mechanisms are acting to ameliorate the seizures.

As we have shown so far, a variety of PS are observed in the peri‐ictal period and may thus affect quality of life. Improving seizures may improve PS, but psychiatrists unaware of the association between PS and seizures may administer standard psychiatric treatments while leaving the incoming epileptic seizures untreated. Understanding the relationship between seizures and PS is thus very important.

This study had several limitations. First, the number of cases in subgroups of PS was small and might have been insufficient to fully elucidate the features of PS. Second, our findings may not be generalizable to all epilepsy patients, because our population represented patients seen in a psychiatry department, and the ratio of patients with IPD and peri‐ictal PS may have been higher than in the general population. Third, accurate assessment of pre‐ictal PS requires symptoms to be recorded at the time of occurrence and the onset of subsequent epileptic seizures to be observed. In this study, accurate identification of any temporal relationship between PS and seizures was not possible because of the dependence on the retrospective recollections of patients and family members. Fourth, while menstruation may influence the onset of seizures or PS, this study did not evaluate the association with menstruation.

Peri‐ictal PS are important for elucidating the pathophysiological relationship between epileptic seizures and PS. Although our study investigated the relationship between pre‐ and post‐ictal PS, further studies (such as a prospective evaluation of peri‐ictal PS) will be needed to clarify PS in epilepsy.

## CONCLUSION

We investigated pre‐ and post‐ictal PS. Fourteen percent of patients showed pre‐ictal PS, 39% had post‐ictal PS, and 10% had both pre‐ and post‐ictal PS. The most frequent PS were irritability symptoms (7%) and anxiety symptoms (5%) in the pre‐ictal period and depressive symptoms (25%) and anxiety symptoms (16%) in the post‐ictal period. The duration of PS was most commonly more than 1 day and less than 1 week. Our study suggests that post‐ictal PS were related to IPD, particularly that post‐ictal irritability symptoms and psychotic symptoms were associated with psychosis in IPD. Further, the number of cases with only post‐ictal PS was significantly higher than the number of cases with only pre‐ictal PS, while cases with pre‐ictal PS were associated with a significantly higher rate of post‐ictal PS occurrence compared to cases without pre‐ictal PS.

## AUTHOR CONTRIBUTIONS

**Shingo Yasumoto**: Conceptualization; data curation; investigation; methodology; project administration; resources; writing—original draft. **Hiromichi Motooka**: Investigation; methodology; resources; supervision; writing—review and editing. **Yuji Ito**: Investigation; resources. **Masaya Mashimoto**: Investigation; resources. **Kenta Murotani**: Formal analysis. **Ryota Ogata**: Formal analysis. **Motohiro Ozone**: Conceptualization.

## CONFLICT OF INTEREST STATEMENT

The authors declare no conflicts of interest.

## ETHICS APPROVAL STATEMENT

This study was conducted at the Kurume University Hospital and approved by the regional Scientific Ethics Committee (approval no. 19165, date of approval: December 6, 2019).

## PATIENT CONSENT STATEMENT

Written informed consent was obtained from all participants in this study or their family members prior to study enrollment.

## CLINICAL TRIAL REGISTRATION

N/A.

## Supporting information

Supporting File 1.

Supporting File 2.

## Data Availability

The data that support the findings of this study are available on request from the corresponding author. The data are not publicly available due to privacy or ethical restrictions.
